# Supplementary material for: Trace elements concentration in blood of nesting Kemp’s Ridley turtles (Lepidochelys kempii) at Rancho Nuevo sanctuary, Tamaulipas, Mexico
Source: PLoS One. 2022 Nov 2;17(11):e0269346. doi: 10.1371/journal.pone.0269346 (PMC9629633; doi:10.1371/journal.pone.0269346)
Supplement: Attachment — Submitted filename: Response.docx [file pone.0269346.s001.docx]

PLOS ONE

PONE-D-22-12917

Determination of heavy metals in blood of nesting Kemp’s Ridley turtles (*Lepidochelys kempii*) at Rancho Nuevo sanctuary, Tamaulipas, Mexico.

Reviewer #1:

**Comments** 
Overall: Good paper, I like that each element analyzed was given context within the paper. Only small details that I would like to be cleared up below.

| **Comments** | Reply |
| --- | --- |
| **Introduction:** It is interesting that the deepwater horizon event was mentioned but the current study is not looking at PAHs. It may be good to mention what common heavy metals are found near oil drilling operations. It is a bit confusing to mention it without measuring those contaminants in these turtles. | Done, the common metals associated with crude oil deposition and spills were added. |
| **Introduction:** The deepwater event was also mentioned in the results but I didn’t quite understand why? To explain that a recent event lowered turtle numbers? That the event affected metal accumulation? Some extra wording may be needed to clarify that. | Done, the mention of the event was to describe the new recruits of Kemp´s Ridley to the adult population. |
| **Results:** Something that I notice was missing in the results was the CRM recovery by element. I think its important that there at least be a table showing the recovery of each element in CRM and/or other QA/QC. | This is presented in methodology at the end of the Trace elements analysis section: the recovery percentage of the analyzed trace elements was between 89-106%.  We consider this information unnecessary as a table in the results section since it is part of the internal controls of the methodology and is presented in the corresponding section. However, we leave it for the Editor's consideration. |
| I am a bit confused about Table 4. The text mentions a relationship between elements which suggest table 4 lists p-values but then says size and metals are not correlated? The table caption should be specific in saying whether this is R value, R2 , or p-value. | According to the statistical analysis realized and described in the methodology section: A simple regression model (*R^2^> 50%)* was performed to find the statistical relationship between the trace elements concentrations and the biometrics.  In the last paragraph of results, it says: “in the present study no relationships were identified, neither between elements analyzed nor between turtle size *vs* trace elements concentration (*R^2^*<50%)”.  However, we consider table 4 unnecessary, so we have deleted it to avoid confusion.  We thank the reviewer for their observation. |

Reviewer #2:

**Comments:** This manuscript describes the concentrations of a variety of “standard” trace elements in the blood of Kemp’s Ridley turtles. While topical and perhaps useful to monitoring the health of this critically-endangered species, the use of analytical methods with inadequate detection limits makes the actual use and interpretation of these data questionable. Indeed, most of my review deals with analytical questions.

| **Comments** | Reply |
| --- | --- |
| In their introduction (p. 2, first paragraph) where they say, “Heavy metals are persistent in the environment as they are not degraded over short periods…” Metals are elements, not organic compounds, so they cannot ever “degrade,” they can only change phases (particulate, dissolved, colloidal), locations (e.g., tissue, sediments, etc.), or chemical forms/speciation. | The sentence was changed for a better redaction: Due to their speciation capacity, trace elements are persistent in the environment due to their speciation capacity |
| The use of the term “heavy metals” is a historic misnaming because some of these are not metals (e.g., As and Se) and many do not have high atomic weights; just call them trace elements, or essential and toxic trace elements. | Done, heavy metals were rewritten as trace elements (toxic and essential) to avoid confusion. |
| My analytical issues with this paper are because the proper choice of collection and storage, sample treatment (digestions), and sample analyses, all affect the data quality, but were not well described or in fact were not adequate.  Sample collection (p. 2). Are the 10 mL tubes plastic and if so, what type? How long were these samples stored under refrigeration? Long storage can affect concentrations due to loses from adsorption to the container walls, or volatilization (Hg, Se). | We omitted a detailed description of this method, since we cite Ley-Quiñónez et al. (2011) paper, where the methodology is described in detail, as indicated in the Trace elements analysis section: “Acid digestions of the blood samples obtained were performed for their determination using methodology previously described”  That said, we are including the method in the manuscript as suggested by the reviewer. This reads as follows:  “Each digestion obtained was measured with deionized water in 25 mL volumetric polypropylene flasks and refrigerated until analysis, which occurred in a period not exceeding 24 h after digestion to avoid volatilization or adsorption by the flask walls.” |
| Metal analysis (p. 4). What is “fortified with standards of reference?” Was this the standard additions method of calibration? What were the blanks, just deionized water analyzed directly, or where there were process blanks that went through all the handling steps? The recovery percentages between 89-106% are not unusual but a table of recoveries for a statistically rigorous assessment of their method’s accuracy for each element is needed. | This information is presented in the second paragraph of the Trace elements analysis section: “Analyzes were performed in duplicate fortified with standards of reference (Perkin Elmer GFAAS Mixed Standard)”.  In reference to the blanks, the manuscript now says: Blanks (deionized water) were placed every eight and underwent the same digestion process to detect possible contamination. |
| Results and discussion. It is not unreasonable to assume that feeding is the main source of trace elements for a higher trophic-level organism (see for example in Luoma and Rainbow’s topical book, Metal Contamination in Aquatic Environments, that covers many of the aspects of this paper). Of their trace elements, Cd, Hg, and Se have probably received the most attention, but the use of an ICP-OES for determining their concentrations is simply useless when their background concentrations are so low to begin with and now you are partitioning into a body fluid. Working near the detection limits means poorer precision and therefore less accuracy. I know that cost may be a factor, but in fact they’re wasting the precious blood and field efforts for simply getting below or near detection limit data. | The results and discussions are based not only on the results, but also with the background on trace metals in the Gulf of Mexico and the areas trophic webs. Food, as mentioned, is the main means that organisms are exposed to trace elements. And previous studies reflect the low concentrations present in the organisms, particularly those that are food for *L. kempii* turtles.  Regarding the analysis of the metals, Cd, Se and Hg, in previous studies by both the authors of this manuscript, as well as other cited authors, high levels of Cd and Se have been found in sea turtles and for this in the use of ICP-OES has proved to be suitable for the analysis. This is the first study of its type in Kemps ridley turtles, and we found that these elements are not present in high levels as in other species (but we could not have known this without doing the analysis) this finding is discussed in the manuscript. For Hg, the detection limit of the analysis equipment is 500 ppb and showed the low levels that occur in Kemps ridley turtles, which is consistent with levels in other species of sea turtles in the Gulf of Mexico. The manuscript does not discuss the levels present in other species but points out that even at lower levels than those presented in our results, Hg can present a sublethal impact on the health of sea turtles. Therefore, we point out in the conclusions the importance of this metal, which even in low concentrations can present an effect at a sublethal level in sea turtles, and that the speciation of Hg should also be considered for future studies. Due to the above, we consider that the scope of this manuscript, being the first study on trace metals in nesting kemps ridley turtles, is relevant and presents new findings. We point out the limitations of the study in the manuscript, and suggest future research, which would help to understand the possible impact that these low concentrations may have on the health of nesting Kemps ridley turtles.  We have taken into account the reviewers comments and believe that we have made the necessary adjustments in the discussions and conclusions. We would like to thank the reviewer for their suggestions and for highlighting where we could improve the clarity of the manuscript. |
| As is not at all like Cd (p. 8) in its chemistry and biochemistry, not to mention its mode of toxicity. And Hg (pp. 8-9) is a critical contaminant element, so having below detection limits data squanders the interpretation of your data and comparing your results to others for the same species or other turtles. The topic of bioconcentration can then be adequately addressed using better methods. | “As is not at all like Cd” was an editing error in the manuscript and has been removed.  About the Hg comment: this is addressed in the reply to the previous reviewer comment. |

JOURNAL REQUIREMENTS

| **Comments** | Reply |
| --- | --- |
| Please ensure that your manuscript meets PLOS ONE's style requirements, including those for file naming. | Done, changes were made. |
| Thank you for stating the following in the Acknowledgments Section of your manuscript:  [Research was authorized by SEMARNAT (permit numbers: SGPA/DGVS/04674/10 and SGPA/DGVS/003769/18) and approved by the national park authority National Commission of Protected Natural Areas (CONANP). The article processing fee was supported by COFAA-IPN. SEMARNAT permit authorizations: SGPA/DGVS/04674/10 and SGPA/DGVS/003769/18. We would like to thank the community of Rancho Nuevo Tamaulipas, Mexico and especially Juan Martínez for his support and to Martha López, director of CONANP Tamaulipas.  Institutional Review Board Statement: The study was conducted according to the guidelines of the Mexican authorities, SEMARNAT to study and manage wildlife samples or species.]  We note that you have provided funding information that is not currently declared in your Funding Statement. However, funding information should not appear in the Acknowledgments section or other areas of your manuscript. We will only publish funding information present in the Funding Statement section of the online submission form.  Please remove any funding-related text from the manuscript and let us know how you would like to update your Funding Statement. | Funding from the manuscript was removed |
|  |  |
